# Ubiquitylation of MFHAS1 by the ubiquitin ligase praja2 promotes M1 macrophage polarization by activating JNK and p38 pathways

**DOI:** 10.1038/cddis.2017.102

**Published:** 2017-05-04

**Authors:** Jing Zhong, Huihui Wang, Wankun Chen, Zhirong Sun, Jiawei Chen, Yajun Xu, Meilin Weng, Qiqing Shi, Duan Ma, Changhong Miao

**Affiliations:** 1 Department of Anesthesiology, Fudan University Shanghai Cancer Center, Shanghai, China; 2 Department of Oncology, Shanghai Medical College, Fudan University, Shanghai, China; 3 Jiangsu Province Key Laboratory of Anesthesiology, Xuzhou Medical College, Xuzhou, China; 4 Department of Anesthesiology, Children’s Hospital of Fudan University, Shanghai, China; 5 Key Laboratory of Metabolism and Molecular Medicine, Ministry of Education, Department of Biochemistry and Molecular Biology, Collaborative Innovation Center of Genetics and Development, Institute of Biomedical Sciences, School of Basic Medical Sciences, Fudan University, Shanghai, China

## Abstract

Sepsis is a systemic inflammation caused by infection. The balance between M1–M2 macrophage polarization has an essential role in the pathogenesis of sepsis. However, the exact mechanism underlying macrophage polarization is unclear. We previously showed that levels of malignant fibrous histiocytoma amplified sequence 1 (MFHAS1) were significantly elevated in septic patients compared with those in nonseptic patients, and involved in the activation of Toll-like receptor (TLR) 2/c-Jun N-terminal kinase (JNK)/nuclear factor (NF)-*κ*B pathway. In the present study, we explored whether MFHAS1 was involved in macrophage polarization and determined the effect of MFHAS1 on inflammation. We performed *in vitro* pulldown assays and *in vivo* co-immunoprecipitation assays and found that E3 ubiquitin ligase praja2 could directly bind to MFHAS1. *In situ* immunostaining analysis confirmed the colocalization of endogenous praja2 with MFHAS1. We first reported that praja2 promotes the accumulation of ubiquitylated MFHAS1 but does not degrade it. Moreover, our results indicate that MFHAS1 ubiquitylation by praja2 positively regulates TLR2-mediated JNK/p38 pathway and promotes M1 macrophage polarization, M2 to M1 macrophage transformation and inflammation.

Sepsis, which is caused by excessive systemic host inflammatory response, is an important reason for patients death in ICU.^1^ Unfortunately there is no effective treatment for sepsis. Therefore, many studies have attempted to identify strategies for treating sepsis. The balance between innate immunity and inflammatory injury is closely associated with sepsis-induced mortality.^2^ As the central regulators of the innate immune system,^3^ macrophages play essential roles in the pathogenesis of sepsis. Based on their microenvironment, macrophages polarize to either M1 or M2 phenotype.^4^ M1 macrophages increase the secretion of pro-inflammatory mediators such as TNF-*α*, IL-6, iNOS and IL-1*β*, and M2 macrophages perform anti-inflammatory functions.^5^ Ly6C expression in macrophages is thought to be correlated with M1 polarization.^6, 7^ Thus, the balance between M1 and M2 macrophage polarization directly affects sepsis-related inflammation.

Sakabe *et al.*^8^ identified a novel gene named malignant fibrous histiocytoma amplified sequence 1 (*MFHAS1*), which is a potential oncogene isolated from malignant fibrous histiocytomas (MFHs).^9^ However, limited information is available about this gene. As the smallest member of ROCO protein family, MFHAS1 has a conserved supra-domain known as ROC-COR.^10^ Results of bioinformatics analysis suggest that MFHAS1 has a role in innate immunity.^10^ Moreover, MFHAS1 has an important role in Toll-like receptor (TLR) 3/4-dependent signaling.^11^ MFHAS1 also participates in erythroid differentiation through Raf/MEK/ERK pathway.^12^ MFHAS1 has a LRR-containing domain, suggesting that MFHAS1 may determine cell fate, and is involved in cellular processes such as apoptosis and ubiquitin-related processes.^11^ Moreover, ROC/GTPase domain of MFHAS1 is critical for regulating its cellular functions.^13^

In our previous studies, we found that MFHAS1 is associated with sepsis and enhances the secretion of inflammatory cytokines IL-6 and TNF-*α*, which are M1 macrophage biomarkers, through TLR2/JNK/NF-*κ*B pathway.^14^ Moreover, TLR2-dependent pathway is required for the polarization of bone marrow-derived macrophages to a M2-like phenotype.^15^ NF-*κ*B activation is needed for the polarization of both M1 and M2 macrophages.^16, 17^ Therefore, we investigated the role of MFHAS1 in macrophage polarization and determined the relationship between the mitogen-activated protein kinase (MAPK) pathway and M1–M2 macrophage polarization balance.^18, 19^

Because of the absence of an effector domain, MFHAS1 must rely on other proteins to control its effector domain.^13^ Results of our mass spectrometry analysis suggest praja ring finger 2 (praja2 also known as PJA2), may interact and form a complex with MFHAS1. Praja2 is an E3 ubiquitin ligase.^20^ Ubiquitin–proteasome system (UPS) is emerging as an important regulator of cell metabolism, growth and survival^21^ and is associated with several diseases. Action of activating enzymes (E1s), conjugating enzymes (E2s) and ligases E3s facilitates ubiquitylation.^22^ E3 ubiquitin ligases, which are classified into two families based on the presence of a HECT or RING domain, have been studied widely.^23^ RING E3 ubiquitin ligases regulate inflammation.^24^ They are also critical regulators of TLRs pathways. Depletion of Casitas B-lineage lymphoma-b (Cbl-b), a RING E3 ubiquitin ligase, promotes IL-6 expression in saturated fatty acids-induced peritoneal macrophages through TLR4-mediated JNK/NF-*κ*B pathway^25^ and induces pro-inflammatory cytokine TNF-*α*, MCP-1 and IL-6 secretion in bone marrow-derived cultured mast cells (BMMCs).^26^ Expression of another RING E3 ubiquitin ligase Pellino-1 activates NF-*κ*B, upregulates TLR2-mediated NF-*κ*B activation and enhances IL-8 secretion.^27, 28^ Emerging researches suggest that E3 ubiquitin ligases are novel targets for regulating TLR pathways and for treating inflammatory diseases. Praja2, a RING E3 ubiquitin ligase, is widely expressed in cells and several tissues and is probably involved in inflammation.^29, 30^ Researches have shown that praja2 ubiquitylates MOB1 to attenuate Hippo signaling and promotes glioblastoma growth.^21^ Praja2 also controls the stability of PKA regulatory subunits and may trigger the MAPK pathway by forming a stable complex with PKA.^31, 32^ A recent study showed that praja2 regulates ERK pathway, affects cell proliferation in cancer cells and affects the differentiation of embryonic stem cells.^33^ Therefore, we hypothesized that praja2 ubiquitylates MFHAS1 to regulate MAPK pathway and the balance of M1–M2 macrophage polarization.

In the present study, we found that praja2 ubiquitylated and interacted with MFHAS1, thus activating the TLR2/JNK/p38/NF-*κ*B pathway and promoting the polarization of macrophages to the M1 phenotype.

## Results

### MFHAS1 forms a complex with praja2

**Figure 1:**
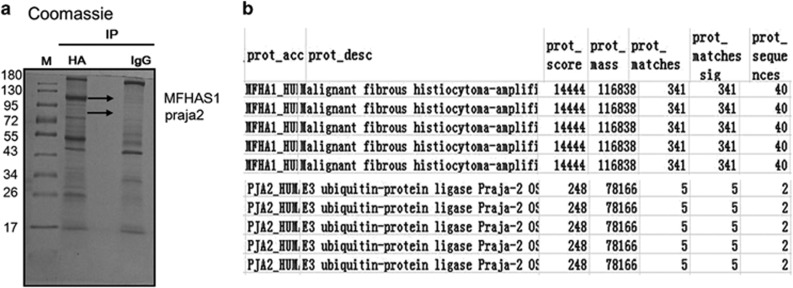
Identification of potential MFHAS1 interactors. (**a**) Identification of MFHAS1-associated proteins through gel-based proteomics. Protein bands not seen in the control sample, were selected for analysis. (**b**) MFHAS1 and praja2 were identified by performing mass spectrometry analysis

**Figure 2:**
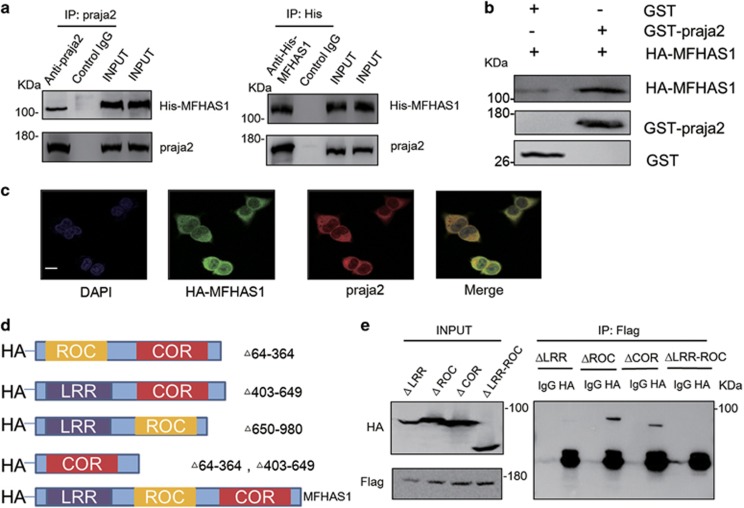
MFHAS1 forms a complex with praja2. (**a**) HEK293 cells were transiently transfected with a plasmid expressing His-tagged MFHAS1. After 24 h of transfection, the cells were treated with MG132 (20 *μ*M) for 6 h and were collected. The cell lysates were immunoprecipitated using anti-praja2 or anti-His antibody. (**b**) HEK293-MFHAS1 cell lysates were used for performing pulldown assays with purified GST or GST-praja2 fusion protein, followed by immunoblotting with anti-GST and anti-HA antibodies. (**c**) HEK293-MFHAS1 cells were subjected to double immunostaining with monoclonal anti-HA and polyclonal anti-praja2 antibodies. The images were collected and were analyzed using a confocal microscope. Magnification of selected areas is shown (insets). Scale bar, 10 *μ*m. (**d**) Schematic representation of MFHAS1 constructs used in this study. (**e**) HEK293 cells were transiently co-transfected with plasmids expressing Flag-praja2 and HA-tagged ΔLRR-MFHAS1 (Δ64-364), ΔROC-MFHAS1 (Δ403-649), ΔCOR-MFHAS1 (Δ650-980) or ΔLRR-ROC-MFHAS1 (Δ64-364,403-649). After 24 h of transfection, the cells were treated with MG132 (20 *μ*M) for 6 h and were lysed for performing immunoprecipitation with anti-Flag antibody

Results of mass spectrometry analysis showed successful immunoprecipitation of MFHAS1. In addition, these results showed additional bands representing possible interactors/binding partners of MFHAS1, which were not obtained for the control group. Praja2 is a possible interactor of MFHAS1 (Figures 1a and b). Next, we conducted co-immunoprecipitation assay and found that exogenous Flag-praja2 and co-expressed His-MFHAS1 formed a stable complex (Figure 2a). We next performed *in vitro* pulldown assays and confirmed that praja2 could directly bind to MFHAS1. Purified glutathione *S*-transferase (GST)-praja2 protein fused with glutathione beads in lysates of HEK293 cells stably expressing HA-MFHAS1 (HEK293-MFHAS1) (Figure 2b). *In situ* immunostaining analysis of HEK293 cells showed partial colocalization of endogenous praja2 with exogenous HA-MFHAS1. Overlapping signals were detected in the perinuclear region and cytoplasm (Figure 2c). Next, we constructed four plasmids to determine the MFHAS1 domain that interacted with praja2 (Figure 2d). Deletion mutagenesis and co-immunoprecipitation assays showed that residues 64-364 (LRR domain) of MFHAS1 interacted with praja2. (Figure 2e).

### Praja2 ubiquitylates but does not degrade MFHAS1

**Figure 3:**
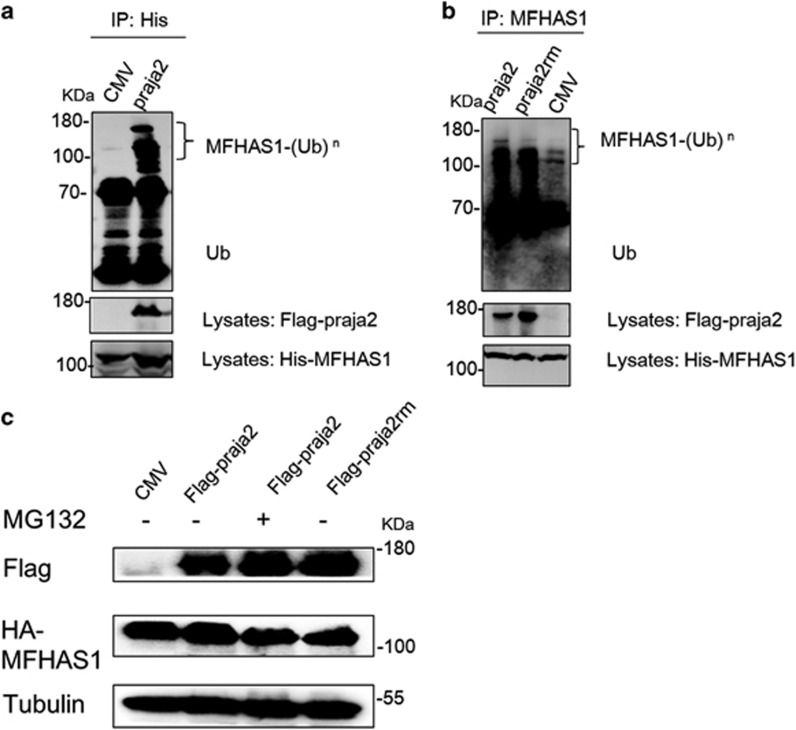
Praja2 ubiquitylates but does not degrade MFHAS1. (**a**) HEK293 cells were transiently transfected with plasmids expressing His-tagged MFHAS1, HA-tagged ubiquitin and Flag-tagged praja2 or with an empty PCDH plasmid. After 24 h of transfection, the cells were treated with MG132 (20 *μ*M) for 6 h and were collected. The cell lysates were immunoprecipitated with anti-His antibody. (**b**) HEK293 cells were transiently transfected with plasmids expressing His-tagged MFHAS1, HA-tagged ubiquitin and Flag-tagged praja2 or Flag-tagged praja2rm or with and an empty PCDH plasmid. After 24 h of transfection, the cells were treated with MG132 (20 *μ*M) for 6 h and were collected. The cell lysates were immunoprecipitated with anti-MFHAS1 antibody. (**c**) HEK293-MFHAS1 cells were transiently transfected with an empty PCDH plasmid and a plasmid expressing Flag-praja2 or Flag-praja2rm. After 24 h of transfection, the cells were treated with or without MG132 (20 *μ*M) for 6 h and were collected. The cell lysates were immunoblotted with the indicated antibodies

We conjectured that the E3 ubiquitin ligase praja2-ubiquitylated MFHAS1 in living cells. We found that Flag-praja2 promoted the accumulation of ubiquitylated forms of His-MFHAS1 (Figure 3a). However, expression of an inactive praja2 mutant (praja2rm) slightly attenuated the ubiquitylation of MFHAS1 compared with that in cells expressing praja2 (Figure 3b). Next, we measured the expression of MFHAS1 in HEK293-MFHAS1 cells transfected with Flag-praja2-expressing plasmid or CMV control plasmid and found that praja2 did not significantly decrease MFHAS1 expression compared with that in control cells. Moreover, we found that treatment with MG132 or transfection with a praja2rm-expressing plasmid did not affect MFHAS1 expression (Figure 3c).

### Ubiquitylation of MFHAS1 by praja2 promotes M1 polarization and inflammation

**Figure 4:**
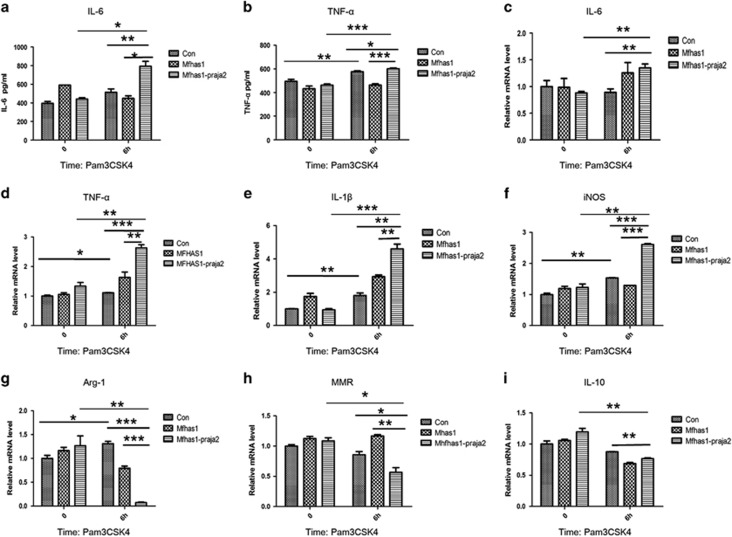
Ubiquitylation of MFHAS1 by praja2 upregulates the expression of M1 macrophage polarization biomarkers and inhibits the expression of M2 macrophage polarization biomarkers. RAW264.7-MFHAS1-praja2 cells were transiently co-transfected with 2.75 *μ*g plasmid expressing HA-MFHAS1 and 2.75 *μ*g plasmid expressing Flag-praja2 plasmids, RAW264.7-MFHAS1 cells were transiently co-transfected with plasmid expressing 2.75 *μ*g HA-MFHAS1 and 2.75 *μ*g empty PCDH plasmid, and control cells were transfected with 5.5 *μ*g empty PCDH plasmid. After 24 h, the cells were treated with or without Pam3CSK4 (10 ng/ml) for 6 h and were collected. Next, secretion of IL-6 and TNF-*α* was detected by performing ELISA (**a** and **b**). The mRNA levels of genes encoding M1 and M2 macrophage biomarkers IL-6, TNF-*α*, IL-1*β*, iNOS, Arg-1, MMR and IL-10 were quantified by performing qPCR and were normalized to the mRNA level of the gene encoding actin. All other groups were calibrated to 0 h control group (**c**–**i**). Values are expressed as means±S.D. of at least four independent experiments. *P<0.05, **P<0.01 or ***P<0.001

Compared with that in RAW264.7-MFHAS1 cells (RAW264.7 cells transfected with HA-MFHAS1-expressing plasmid) and control cells, secretion of IL-6 and TNF-*α* was significantly elevated in RAW264.7-MFHAS1-praja2 cells (RAW264.7 cells transfected with HA-MFHAS1 and Flag-praja2-expressing plasmids) after 6 h Pam3CSK4 (TLR2 ligand) treatment (*P*<0.05; Figures 4a and b). We next examined the mRNA levels of genes encoding M1 macrophage biomarkers IL-6, TNF-*α*, IL-1*β* and iNOS by performing qPCR. We found that praja2 significantly increased the mRNA levels of genes expressing IL-6, TNF-*α*, IL-1*β* and iNOS (*P*<0.05; Figures 4c and f). However, mRNA levels of genes encoding M2 macrophage biomarkers Arg-1, MMR and IL-10 were significantly decreased in RAW264.7-MFHAS1-praja2 cells compared with those in RAW264.7-MFHAS1 and control cells (*P*<0.05; Figures 4g and i). Levels of M1 and M2 macrophage biomarkers did not differ significantly between RAW264.7-MFHAS1-praja2 cells and control cells without TLR2 stimulation (*P*>0.05). Moreover, we transfected RAW264.7 cells with shRNA against *MFHAS1* to suppress its expression. We found that TLR2 stimulation only did not induce all M1-like phenotypes as what has been found in RAW264.7-MFHAS1 cells and RAW264.7-MFHAS1-praja2 cells (Supplementary Figure 1).

**Figure 5:**
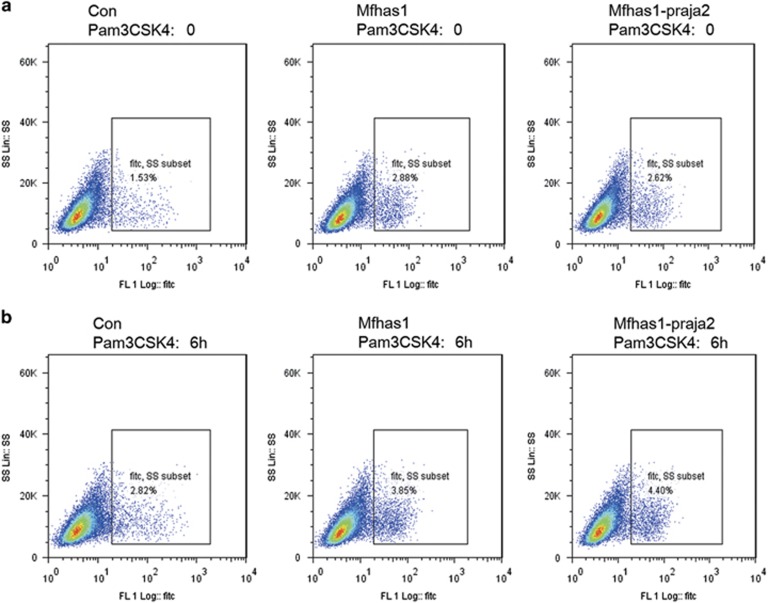
Ubiquitylation of MFHAS1 by praja2 upregulates Ly6C expression in RAW264.7 cells. The percentage of Ly6C^+^ RAW264.7-MFHAS1-praja2, RAW264.7-MFHAS1 and RAW264.7-Con cells was analyzed by performing flow cytometry analysis at 0 (**a**) and 6 h (**b**) after Pam3CSK4 treatment. The cells were prepared as described in the ‘Materials and Methods’ section and were stained with anti-Ly6C antibody. The experiment was repeated three times and similar results were obtained

We also determined Ly6C expression in RAW264.7-MFHAS1-praja2, RAW264.7-MFHAS1 and RAW264.7-Con cells by performing flow cytometry analysis and found that Ly6C expression was upregulated in RAW264.7-MFHAS1-praja2 cells after 6 h Pam3CSK4 treatment (Figures 5a and b).

### Ubiquitylation of MFHAS1 activates JNK and p38 pathways and NF-*κ*B production through TLR2 stimulation

**Figure 6:**
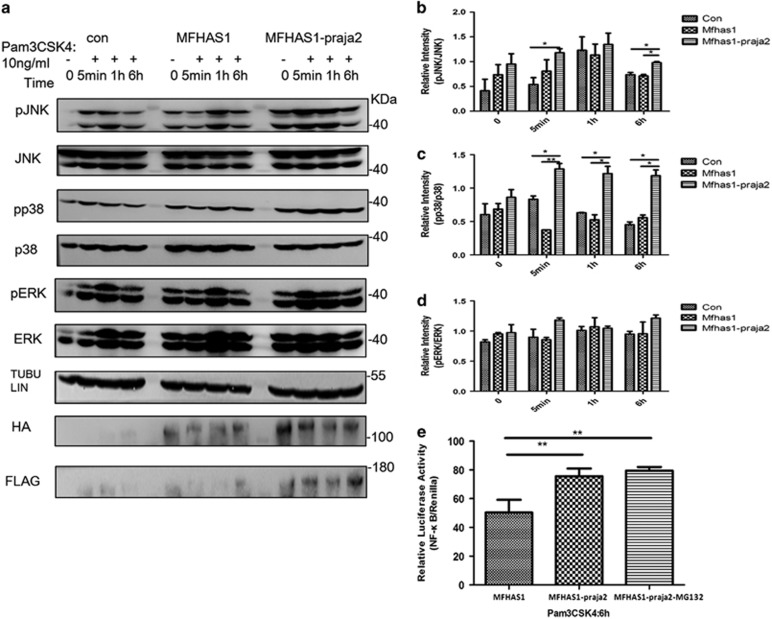
Ubiquitylation of MFHAS1 by praja2 activates JNK/p38 and NF-*κ*B production through TLR2 activation. (**a**) RAW264.7-MFHAS1-praja2 cells were transiently co-transfected with 2.75 *μ*g plasmid expressing HA-MFHAS1 and 2.75 *μ*g plasmid expressing Flag-praja2 plasmids, RAW264.7-MFHAS1 cells were transiently co-transfected with plasmid expressing 2.75 *μ*g HA-MFHAS1 and 2.75 *μ*g empty PCDH plasmid, and control cells were transfected with 5.5 *μ*g empty PCDH plasmid. After 24 h of transfection, the cells were treated with 10 ng/ml Pam3CSK4 for 0, 5 min, 1 h and 6 h. Next, the cells were collected and the protein levels of JNK, p38, ERK and phosphorylated JNK (pJNK), phosphorylated p38 (pp38) and phosphorylated ERK (pERK) were determined by performing western blotting. (**b**–**d**) Band intensities on western blots were semi-quantified using Image J software. (**e**) HEK293 cells were transiently transfected with 100 ng NF-*κ*B luciferase reporter plasmid, 10 ng *Renilla* plasmid and 50 ng plasmid expressing HA-MFHAS1 along with 50 ng empty PCDH plasmid (‘MFHAS1’) or 50 ng plasmid expressing Flag-praja2 (‘MFHAS1-praja2’, ‘MFHAS1-praja2 (MG132)’). After 24 h of transfection, the cells were exposed to 100 ng/ml Pam3CSK4 for 6 h. Next, ‘MFHAS1-praja2 (MG132)’ cells were treated with MG132 (20 *μ*M) for 6 h and were collected. Luciferase activity was measured using the Dual Luciferase kit. Relative luciferase activity was calculated using the ratio of NF-*κ*B (firefly) luciferase activity to *Renilla* luciferase activity. Values are expressed as means±S.D. of at least four independent experiments. *P<0.05, **P<0.01

The MAPK pathway is activated by several receptors, including TLRs, integrins and ion channels, which transduce a signal to adaptors that eventually activate Raf, MEK1/2 and JNK/p38/ERK, the core components of the pathway. We postulated that praja2 regulated JNK/p38/ERK phosphorylation and affected TLR cascade by affecting the role of MFHAS1 in TLR pathway. We examined this by monitoring JNK/p38/ERK phosphorylation in RAW264.7 cells (Figure 6a). At 1 h after TLR2 stimulation, JNK and p38 phosphorylation increased by 1.5-fold compared with their baseline levels in RAW264.7-MFHAS1-praja2 cells. Moreover, levels of phosphorylated JNK (pJNK) were significantly higher in RAW264.7-MFHAS1-praja2 cells compared with those in control cells at 5 min and 6 h after TLR2 stimulation (*P*<0.05; Figure 6b). Levels of phosphorylated p38 (pp38) were significantly higher in RAW264.7-MFHAS1-praja2 cells compared with those in control cells at 5 min, 1 h and 6 h after TLR2 stimulation (*P*<0.05; Figure 6c). However, ERK phosphorylation did not increase compared with its baseline level (Figure 6d). Moreover, praja2 stimulated the luciferase activity of NF-*κ*B in RAW264.7-MFHAS1-praja2 cells after 6 h of Pam3CSK4 stimulation (Figure 6e). Praja2 activated the transcription factor NF-*κ*B by the ubiquitylation of MFHAS1.

### Blocking JNK or p38 pathway or both affects M1 and M2 macrophage polarization

**Figure 7:**
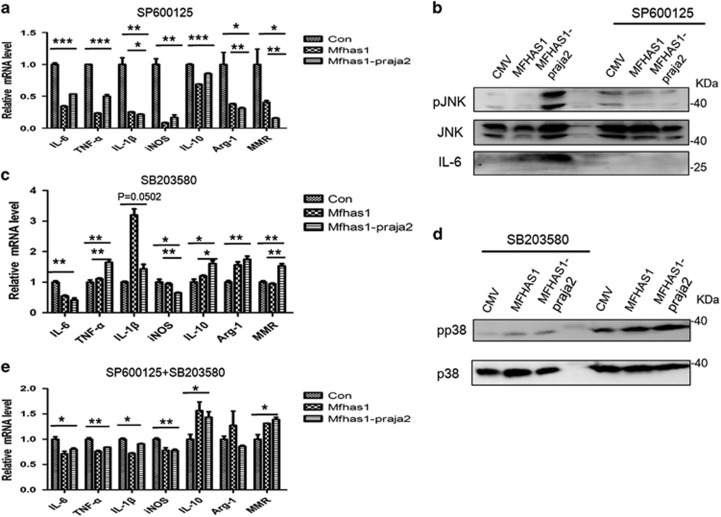
Blockade of the JNK and p38 pathways affects M1–M2 macrophage polarization. RAW264.7 cells were transiently transfected with plasmids expressing HA-tagged MFHAS1 and Flag-tagged praja2 (‘MFHAS1-praja2’) or HA-tagged MFHAS1 (‘MFHAS1’) or with the empty PCDH plasmid (‘CMV’). After 24 h of tranfection, the cells were serum starved and were treated with SP600125 (10 *μ*M; **a** and **b**) or SB203580 (20 *μ*M; **c** and **d**) or both (**e**) for 1 h. Next, the cells were washed and treated with Pam3CSK4 (10 ng/ml) for 6 h in DMEM containing 10% FBS. The mRNA levels of genes encoding M1 macrophage biomarkers (IL-6, TNF-*α*, IL-1*β* and iNOS) and M2 macrophage biomarkers (ARG-1, MMR and IL-10) were quantified by performing qPCR and were normalized using the mRNA level of the gene encoding actin (**a**, **c** and **e**). The protein levels of JNK, pJNK, p38, pp38 and IL-6 were determined by performing western blotting (**b** and **d**). Values are expressed as means±S.D. of at least four independent experiments. *P<0.05, **P<0.01 or ***P<0.001

To investigate the function of the JNK pathway in macrophage polarization, we blocked it by using SP600125 (10 *μ*M). We found blockade of the JNK pathway significantly decreased the expression of M1 macrophage polarization biomarkers in RAW264.7-MFHAS1-praja2 cells compared with that in control cells (*P*<0.05). However, blockade of the JNK pathway did not influence ubiquitylated MFHAS1’s effect on the expression of M2 macrophage polarization biomarkers (Figure 7a). We confirmed the efficiency of SP600125 for blocking the JNK pathway by performing western blotting (Figure 7b). Our results suggested that the JNK pathway only regulated M1 polarization in RAW264.7-MFHAS1-praja2 cells. Similarly, we blocked the p38 pathway by using SB203580 (20 *μ*M). We found that blockade of the p38 pathway increased the expression of M2 macrophage polarization biomarkers and decreased the expression of some M1 macrophage polarization biomarkers in RAW264.7-MFHAS1-praja2 cells compared with that in control cells (*P*<0.05; Figure 7c). We confirmed the efficiency of SB203580 for blocking the p38 pathway by performing western blotting (Figure 7d). These data indicate that the p38 pathway is involved in both M1 and M2 macrophage polarization. Next, we blocked both the JNK and p38 pathways and found that secretion of M1 macrophage polarization biomarkers IL-6, TNF-*α*, IL-1*β* and iNOS decreased, whereas that of M2 macrophage polarization biomarkers increased in RAW264.7-MFHAS1-praja2 cells compared with that in control cells (*P*<0.05; Figure 7e).

These results suggest that the ubiquitylation of MFHAS1 by praja2 has a vital role in M1 macrophage polarization and promotes the transformation of M2 macrophages to M1 macrophages through both the JNK and p38 pathways.

## Discussion

Here, we report the mechanism of signal strengthening during macrophage polarization involving the TLR2 pathway, which is adjusted by MFHAS1 and praja2. We found that praja2 could directly bind to MFHAS1 and ubiquitylate it. Modification of MFHAS1, that is, its ubiquitylation by praja2, activated the TLR2/JNK/p38/NF-*κ*B pathway, resulting in M1 macrophage polarization and M2 to M1 macrophage transformation.

Praja2, an E3 ubiquitin ligase, is a novel cancer-associated protein whose expression is upregulated in high-grade glioma.^21^ In the present study, we identified MFHAS1 as a critical praja2 substrate at the molecular level. Furthermore, our result indicates that praja2 ubiquitylates but does not degrade MFHAS1. Protein substrates can be mono-ubiquitylated, multi-ubiquitylated, poly-ubiquitylated and linearly ubiquitylated. Mono- and multi-ubiquitylation change protein interactions, localization and function, but do not degrade the substrate protein. Poly-ubiquitin chains contribute to proteasomal degradation through lysine-48 linkage, whereas linearly ubiquitin chains, the lysine-63 linkage, convert substrate protein into a scaffold during cellular signaling.^34^ Our results suggest that MFHAS1 may be multi-ubiquitylated by praja2. In our subsequent studies, we wish to confirm the modality of ubiquitylated MFHAS1.

Macrophages are key modulator and effector cells in innate immune response, and achieve particular phenotypic characteristics in specific microenvironments, that is, M1-type classical activation and M2-type alternative activation. Multiple diseases are caused by an imbalance in M1–M2 macrophage polarization. However, molecular mechanisms regulating the polarization of macrophages are still unclear.

We first detected the effects of the ubiquitylation of MFHAS1 by praja2 on the expression of M1 and M2 macrophage polarization biomarkers by performing qPCR or ELISA after Pam3CSK4 treatment. We found that, under Pam3CSK4 treatment, MFHAS1 ubiquitylation increased the expression of M1 macrophage biomarkers and decreased the expression of M2 macrophage biomarkers. Results of flow cytometry analysis also showed that the ubiquitylation of MFHAS1 by praja2 upregulates Ly6C, a biomarker of inflammatory cells. As the expression of Ly6C in macrophages is also thought to be correlated with M1 polarization,^6^ these results taken together suggest that ubiquitylated MFHAS1 promotes inflammation and affects the polarization of macrophages, by promoting them to M1 polarization. Activated M1 macrophages kill pathogens and tumor cells while M2 macrophages inhibit inflammatory response, improve tissue repair after inflammation or injury, and shield tumor immune surveillance.^35^ Because M2 macrophages are inflammation-suppressive, transformation of M2 macrophages to M1 macrophages, as that observed in the present study, indicates that ubiquitylated MFHAS1 exerts inflammation-stimulating and tumoricidal effects on macrophages.

The essence of sepsis is inflammation. TLR signaling acts synergistically in the initiation of the innate immune response to bacterial infection during sepsis. TLR2 and TLR4, which are expressed on the cell surface, are the only TLRs known to be responsive to microbial ligands.^36^ TLR4 and TLR2 pathways are the key pathways in sepsis pathophysiology.^37^ Unlike TLR4 and TLR3, TLR2 is associated with deleterious systemic inflammation, cardiac dysfunction, acute kidney injury (a common entity in critically ill patients) and is mostly triggered by severe sepsis.^38, 39, 40^ Our previous study investigated the effect of MFHAS1 on the TLR2 pathway, and determined the difference between TLR2 and TLR4.^14^ In the present study, we investigated M1/M2 macrophage polarization through TLR2 pathway. We used Pam3CSK4, a specific ligand for TLR2, to stimulate the TLR2 pathway in RAW264.7 cells.

**Figure 8:**
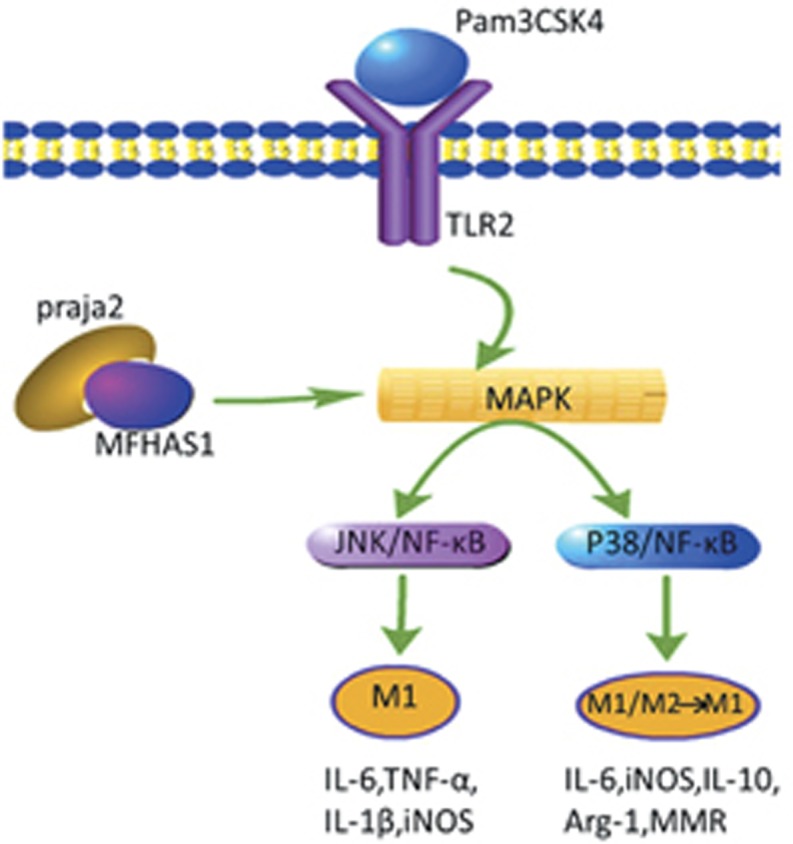
Proposed model of the effect of MFHAS1 ubiquitylation on the MAPK pathway during macrophage polarization. MFHAS1 modification, that is, ubiquitylation by praja2, activates the TLR2/JNK/p38/NF-*κ*B pathway, resulting in M1 macrophage polarization and M2 to M1 macrophage transformation

The MAPK pathway is reported to be under TLR2 signaling cascade. Several studies have shown that the MAPK pathway is involved in the UPS.^41, 42^ To our knowledge, many pathways are involved in macrophage polarization, including MAPK, JAK–STAT6 and PI3K/AKT pathways.^43, 44, 45^ The MAPK pathway is activated druing the polarization of macrophages and expression of relevant inflammatory mediators.^43, 46^ NF-*κ*B, which is also associated with adaptive immunity and inflammation, is the main regulator of the UPS.^47^ Recent studies have shown that ubiquitin is involved in the degradation of NF-*κ*B, and inhibitor I*κ*B, processing of NF-*κ*B precursors and activation of I*κ*B kinase through a degradation-independent mechanism.^48^ NF-*κ*B is the central transcription factor that promotes pro-inflammatory as well as anti-inflammatory processes. Moreover, NF-*κ*B may promote macrophages to immunosuppressive phenotype to limit their tumoricidal and bactericidal functions.^49^ Our previous study showed that MFHAS1 activated the TLR2/JNK/NF-*κ*B pathway. Therefore, we assessed the effects of ubiquitylated MFHAS1 on JNK/p38/ERK phosphorylation in the present study. We found that the ubiquitylation of MFHAS1 induced the phosphorylation of JNK/p38 but not of ERK. In addition, we performed luciferase assay to determine the transcriptional activity of NF-*κ*B, the downstream transcription factor of TLR2. Results of the luciferase assay showed that praja2 activated NF-*κ*B by ubiquitylating MFHAS1. Next, we used specific inhibitors SP600125 and SB230580 to block the JNK and p38 pathways, respectively. We found that the JNK pathway regulated the secretion of M1 macrophage biomarkers (IL-6, TNF-*α*, IL-1*β* and iNOS), and that the p38 pathway regulated the expression of both M1 and M2 biomarkers (IL-6, iNOS, ARG-1, MMR and IL-10). Thus, the effects of praja2-ubiquitylated MFHAS1 on M1/M2 macrophage polarization and inflammation can be summarized as follows: the ubiquitylation of MFHAS1 positively regulates signaling pathways downstream of the TLR2 pathway, the JNK and p38 pathways, resulting in the macrophages transformation from M2 to M1, thus stimulating inflammation (Figure 8).

In conclusion, our data indicate that the E3 ubiquitin ligase praja2 ubiquitylates MFHAS1 and promotes M1 macrophage polarization and transformation from M2 to M1 by activating the JNK and p38 pathways. MFHAS1 can be ubiquitylated by praja2 and the ubiquitylation of MFHAS1 has an important role in the M1 macrophage transformation of RAW264.7 cells through TLR2-mediated JNK and p38 pathways. Our results identify praja2 as a novel inflammation-associated protein whose expression together with that of MFHAS1 predicts the aggressive potential of inflammation. The TLR2/JNK/P38/NF-*κ*B pathway, which is positively regulated by praja2, together with MFHAS1 constitutes a UPS-driven signaling circuit. This circuit influences the polarization of macrophages and stimulates inflammation. We expect that these results will help design more effective target-oriented therapeutic strategies for treating excessive inflammation in patients with sepsis.

## Materials and methods

### Cell culture

Human embryonic kidney cell line (HEK293) that stably expressed HA-tagged MFHAS1 (HEK293-MFHAS1 for short) was kindly gifted by Professor Miao (Fudan University Shanghai Cancer Center, China). RAW264.7 cell line was kindly provided by Professor Ma (Fudan University Shanghai Medical School). All the cell lines were cultured at 37 °C in a 5% CO_2_ incubator and were maintained in DMEM (HyClone, Thermo, Waltham, MA, USA) supplemented with 10% FBS (HyClone) and penicillin and streptomycin.

### Reagents and plasmids

MG132 and p38 pathway blocker SB203580 were purchased from Selleckchem (Houston, TX, USA). JNK pathway blocker SP600125 was purchased from Sigma-Aldrich (St. Louis, MO, USA). Lipofectamine 3000 and Lipofectamine 2000 were purchased from Invitrogen (Carlsbad, CA, USA). Anti-MFHAS1 antibody (sc-390556, 1:500 dilution for immunoblotting and 1:50 dilution for immunoprecipitation) and anti-ubiquitin (anti-Ub) antibody (sc-8017; dilution, 1:500) were purchased from Santa Cruz Biotechnology (Santa Cruz, CA, USA). Anti-praja2 antibody (1:2000 dilution for immunoblotting and 1:100 dilution for immunostaining) was purchased from Bethyl Laboratories (Montgomery, TX, USA). Anti-p38 (8690 P; dilution, 1:1000), anti-pp38 (4511 P; dilution, 1:1000), anti-JNK (9258 P; dilution, 1:1000), anti-pJNK (4668 P; dilution, 1:1000), anti-ERK (4695 P; dilution, 1:1000), anti-phosphorylated ERK (4370 P; dilution, 1:1000) mAbs were provided by Cell Signaling Technology (Danvers, MA, USA). Anti-tubulin mAb (dilution, 1:5000) and anti-Flag mAb (M20008; 1:5000 dilution for immunoblotting and 1:100 dilution for immunoprecipitation) and anti-His mAb (M20001; 1:5000 dilution for immunoblotting and 1:100 dilution for immunoprecipitation) were purchased from Abmart (Shanghai, China). Anti-HA mAb (66006-1-Ig; 1:5000 dilution for immunoblotting and 1:500 dilution for immunostaining) was purchased from Proteintech (Chicago, IL, USA). Dual Luciferase Reporter Assay System (Promega, Madison, WI, USA) was generously gifted by Professor Miao (Fudan University Shanghai Cancer Center, China). Plasmid expressing Flag-praja2 was purchased from GeneChem (Shanghai, China). Plasmid HA-tagged Ub was gifted by Professor Ma (Fudan Unversity Shanghai Medical School). Plasmids expressing HA-tagged MFHAS1, His-tagged MFHAS1 and an empty PCDH plasmid were kindly given by Professor Miao (Fudan University Shanghai Cancer Center, China).

### Western blotting

The clls in each experimental group were lysed in lysis buffer (Beyotime, Shanghai, China) containing protease inhibitors (PMSF; Dingguo, Beijing, China). Proteins obtained were separated by performing SDS-PAGE on a 10% gel, were transferred onto Hybond TM-P membrane (GE Healthcare, Little Chalfont, UK) and were blocked with 8% skimmed milk in TBST (20 mM Tris HCl (pH 8.0), 150 mM NaCl and 0.05% Tween 20) for 1 h at room temperature. Next, the membranes were incubated overnight at 4 °C with appropriate primary antibodies. After washing three times with TBST, the membranes were incubated with peroxidase-conjugated secondary antibodies for 1 h. After washing, the blots were treated with a chemiluminescent reagent (Merck Millipore, Billerica, MA, USA) and were exposed to X-ray films (MidSci, St. Louis, MO, USA). Protein bands obtained were quantified using Image J software (NIH, Bethesda, MD, USA).

### Immunoprecipitation assay

Co-immunoprecipitation experiments were performed to detect the interaction between MFHAS1 (His-tagged) and praja2 (Flag-tagged). For this, similar amounts of proteins were incubated overnight at 4 °C with 4 *μ*l praja2 or anti-His antibodies or normal IgG (control) and were precipitated with 30 *μ*l protein G-Agarose (Roche, Switzerland) for 2 h at 4 °C. The precipitated complexes were washed three times in lysis buffer. Eluted samples were boiled for 10 min in SDS sample buffer, were separated by performing SDS-PAGE on a 10% gel and were transferred onto Hybond TM-P membrane. The membranes were analyzed by performing immunoblotting to detect coprecipitated proteins.

### Mass spectrometry analysis

For mass spectrometry analysis, HEK293-MFHAS1 cells were lysed and precipitated with anti-HA antibody or normal mouse IgG along with protein G-Agarose, as mentioned previously (immunoprecipitation assay). The precipitated complexes were washed and separated by performing SDS-PAGE on a 10% gel before staining with Coomassie Brilliant blue for 3–5 h. The gels were decolorized overnight and were excised. Mass spectrometry analysis was performed in MS analysis room of the Biomedical Research Institute of Fudan University.

### Immunofluorescence assay

After culturing in a 3.5 cm dish for 24 h, HEK293 cells were transiently transfected with a plasmid expressing Flag-praja2. The cells were fixed with fresh acetone-methylalcohol (1:1) solution for 2 min at room temperature, were washed three times in 1 × phosphate-buffered saline (PBS) and were incubated in 5% normal fetal calf serum (blocking solution; HyClone, USA) in a humidified chamber for 1 h at room temperature. Next, the cells were washed three times with 1 × PBS. After incubation with the specific primary antibody diluted in 5% FBS (anti-HA antibody (dilution, 1:500) and anti-praja2 antibody (dilution, 1:100)) overnight at 4 °C, the cells were washed three times in 1 × PBS and were stained with appropriate fluorescein isothiocyanate (FITC)-conjugated (Dingguo, Beijing, China) and tetraethyl rhodamine isothiocyanate-tagged (CWBIO, Beijing, China) secondary antibodies in a humidified chamber for 1 h at room temperature. Nuclei were counterstained with 1 mg/ml 4′,6-diamidino-2-phenylindole dihydrochloride (Sigma, St. Louis, MO, USA). Next, the cells were washed three times in 1 × PBS and were visualized using a confocal microscope (TCS SP8; Leica, Wetzlar, Germany). Image J software was used for images analysis.

### GST pulldown assay

For GST pulldown assay, 13.3 *μ*l glutathione Sepharose TM 4B beads (GE Healthcare) were washed three times in 0.5 ml binding buffer (20 mM Na3PO4, 0.5 M NaCl and 30 mM imidazole (pH 7.4)) and were centrifuged at 1000 × *g* for 5 min each. GST polypeptide hybrid proteins (GST-praja2 or GST; 10 *μ*l) were purified by performing affinity chromatography (Proteintech) and were immobilized on glutathione beads. After incubation for 2 h, the beads were centrifuged at 1000 × *g* 4 °C for 5 min and were incubated overnight at 4 °C with the lysates of HEK293-MFHAS1 cells. The complexes were washed at least three times with binding buffer, and the precipitated proteins were analyzed by performing western blotting as described previously.

### Reporter gene assay

The HEK293 cells were plated in 24-well plates and were co-transfected with 10 ng *Renilla* plasmid (internal transfection control), 100 ng pGL4.32[*luc*2P/NF-*κ*B–RE/Hygro] reporter plasmid (firefly luciferase), 50 ng HA-MFHAS1-expressing plasmid, 50 ng Flag-praja2-expressing plasmid (‘praja2’, ‘praja2 (MG132)’), or empty PCDH plasmid and 50 ng HA-MFHAS1-expressing plasmid (Con) by using Lipofectamine 2000 (Invitrogen). After 24 h of transfection, the cells were treated with 100 ng/ml Pam3CSK4 for 6 h. Next, firefly and *Renilla* luciferase activities were measured for determing NF-*κ*B activation by using Dual Luciferase Reporter Assay System.

### qPCR analysis

Total RNA was extracted from RAW264.7 cells by using Trizol (Invitrogen). The RNA was reverse transcribed using iScript cDNA Synthesis Kit (Bio-Rad, Hercules, CA, USA). cDNA obtained was amplified using primers specific for genes encoding macrophage polarization biomarkers. Quantitative PCR (qPCR) was performed using iTaq SYBR Green Supermix (Bio-Rad) and StepOnePlus Real-Time PCR System (Life Technologies, Carlsbad, CA, USA), according to the manufacturer’s protocol.

### Flow cytometry analysis

RAW264.7-MFHAS1-praja2, RAW264.7-MFHAS1 or control cells were treated with or without 10 ng/ml Pam3CSK4 for 6 h. Next, the cells were collected and were stained for cell surface Ly6C conjugated with FITC (BD Biosciences, Franklin Lakes, NJ, USA) for 30 min at room temperature, according to the manufacturer’s instructions. Next, the cells from all the groups were collected and were analyzed at each time point on the same day by using the same cytometer settings. Surface molecule expression was assessed by determining the percentage of positively stained cells. Flow cytometric data were acquired using a FACSCalibur flow cytometer (BD Biosciences) and were analyzed using FlowJo software (TreeStar Inc., Ashland, OR, USA).

### Statistical analysis

Statistical analysis was performed using *t*-test, and one or two-way ANOVA with GraphPad Prism Version 5 (GraphPad Software, San Diego, CA, USA). Results are reported as mean±S.D. Difference was considered statically significant at *P*<0.05.
