# Correction: Ubiquitylation of MFHAS1 by the ubiquitin ligase praja2 promotes M1 macrophage polarization by activating JNK and p38 pathways

**DOI:** 10.1038/s41419-018-0828-y

**Published:** 2018-07-16

**Authors:** Jing Zhong, Huihui Wang, Wankun Chen, Zhirong Sun, Jiawei Chen, Yajun Xu, Meilin Weng, Qiqing Shi, Duan Ma, Changhong Miao

**Affiliations:** 1 Department of Anesthesiology, Fudan University Shanghai Cancer Center, Shanghai, China; 2 Department of Oncology, Shanghai Medical College, Fudan University, Shanghai, China; 3 Jiangsu Province Key Laboratory of Anesthesiology, Xuzhou Medical College, Xuzhou, China; 4 Department of Anesthesiology, Children’s Hospital of Fudan University, Shanghai, China; 5 Key Laboratory of Metabolism and Molecular Medicine, Ministry of Education, Department of Biochemistry and Molecular Biology, Collaborative Innovation Center of Genetics and Development, Institute of Biomedical Sciences, School of Basic Medical Sciences, Fudan University, Shanghai, China

Correction to: *Cell Death & Disease*; 10.1038/cddis.2017.102; published online 4 May 2017.

The PDF and HTML versions of the article have been updated to include the Creative Commons Attribution 4.0 International License information.

