# Supplementary figures and images for: Ubiquitylation of MFHAS1 by the ubiquitin ligase praja2 promotes M1 macrophage polarization by activating JNK and p38 pathways
Source: Cell Death Dis. 2017 May 4;8(5):e2763–. doi: 10.1038/cddis.2017.102 (PMC5520684; doi:10.1038/cddis.2017.102)

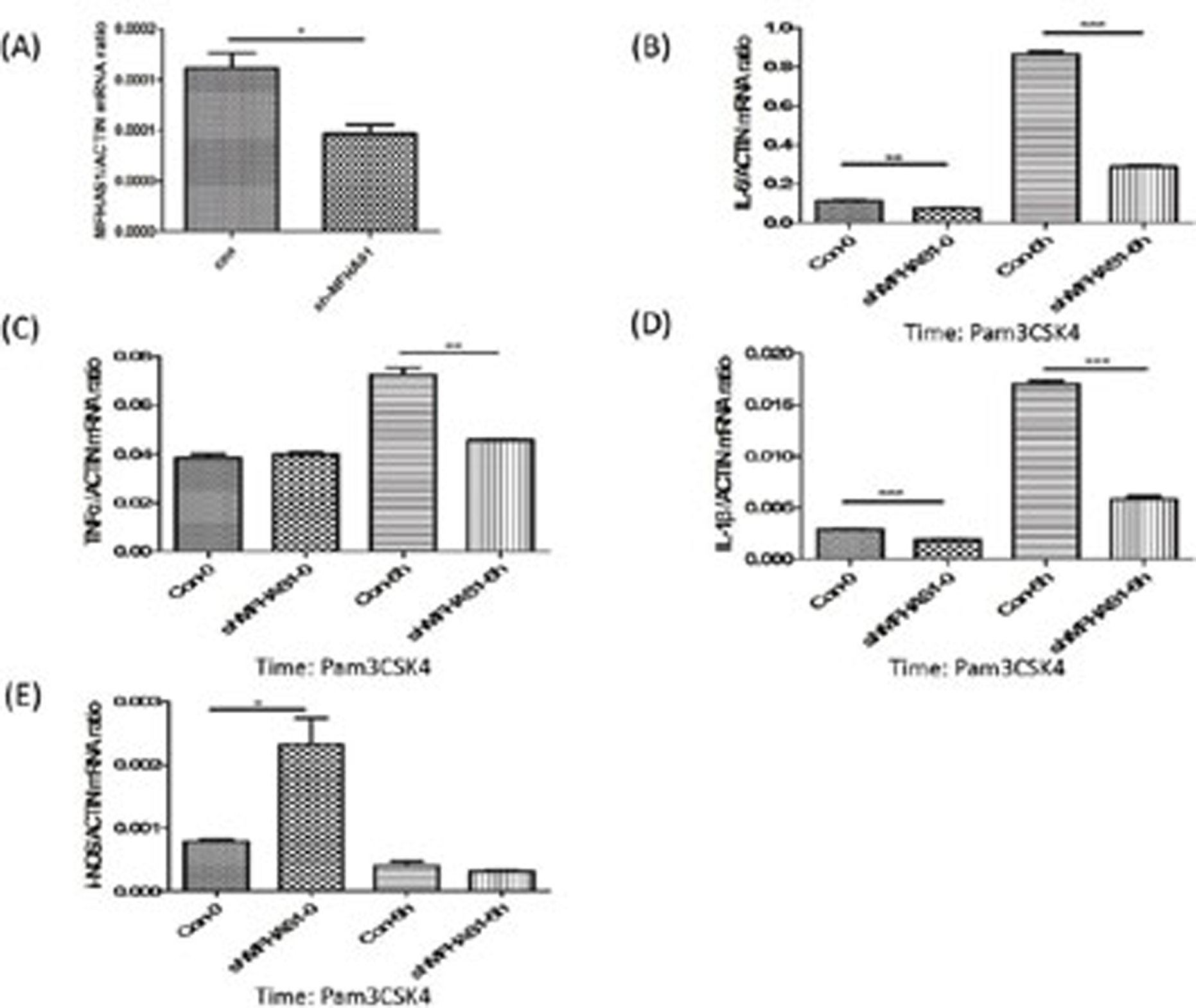

Supplement: Supplementary Figure [file cddis2017102x1.tif]
