# Supplementary material for: Ubiquitylation of MFHAS1 by the ubiquitin ligase praja2 promotes M1 macrophage polarization by activating JNK and p38 pathways
Source: Cell Death Dis. 2017 May 4;8(5):e2763–. doi: 10.1038/cddis.2017.102 (PMC5520684; doi:10.1038/cddis.2017.102)
Supplement: Supplementary Figure Legend [file cddis2017102x2.docx]

**Supplemented figure 1.** RAW264.7 cells were transfected with sh*MFHAS1* or Con plasmid. After 24 h of transfection, the cells were exposed to 10 ng/ml Pam3CSK4 for 6 h. The knockdown efficiency of sh*MFHAS1* (A), and the mRNA levels of M1 macrophage biomarkers (IL-6, TNF-α, IL-1β, and iNOS) were quantified by performing qPCR and were normalized to the mRNA levels of actin (B-E).
